# United Kingdom Co-ordinating Committee on Cancer Research (UKCCCR) Strategy Group Workshop

**DOI:** 10.1038/sj.bjc.6690001

**Published:** 1999-09-24


*British Journal of Cancer* (1999) **79**(1), 2–3
© 1999 Cancer Research Campaign

Workshop review

### United Kingdom Co-ordinating Committee on Cancer Research (UKCCCR) Strategy Group Workshop

### Improvements in patient access to new anti-cancer medicines

The Strategy Group of the United Kingdom Co-ordinating Committee on Cancer Research (UKCCCR) held a workshop on 20 November 1997 to consider the question of 'Improvements in Patient Access to New Anti-Cancer Medicines'. The Chairman was Professor Sir William Asscher (Chairman, UKCCCR) and the meeting was attended by representatives of the Medical Research Council (MRC), Cancer Research Campaign (CRC), Imperial Cancer Research Fund (ICRF) and Leukaemia Research Fund (LRF), together with a number of invited speakers and rapporteurs.

Dr Peter Twentyman (Executive Secretary, UKCCCR) in introducing the Workshop expressed the view that there was, among oncologists in the UK, a large amount of goodwill and enthusiasm for clinical trials, but a strong feeling that a considerable obstacle course lay in the path of those wishing to participate. The steps involved in getting a trial funded and running are widely regarded as excessively burdensome and getting worse. Even when trials are open to accrual, heterogeneity with respect to ethical approval and local funding of support costs has proved problematic. New arrangements for National Health Service (NHS) support of clinical trials will, however, operate from April 1998 and the impact of these will be closely monitored. Difficulties have arisen when company-sponsored trials have competed for accrual with academic-based trials including quality of life and health economic assessment. This could result in difficulties for the determination of cost-effectiveness of new therapies. There have been various recent examples of cancer service providers being unable or unwilling to meet the costs of expensive new anti-cancer treatments.

Professor Paul Workman (Institute of Cancer Research, Sutton) described the importance of collaboration between academia and industry in terms of new drug development. He discussed the commercial issues driving R&D strategy within the pharmaceutical industry and the need to align research to commercial objectives. He went on to describe recent advances in drug discovery and the need for speed in the development of innovative agents. Professor Lewis Smith (MRC Toxicology Unit, Leicester) believed that one of the most important blocks to getting new drugs through the system is getting the drug into man in the first place. He emphasized the need for early 'proof of principle' and he believed that there was a need for a more pragmatic and rational approach to new anti-cancer drugs.

Professor Michael Rawlins [Chairman, Committee on Safety of Medicines (CSM)] explained that, in his view, there was a tendency for regulatory guidelines to examine the past rather than the future. He thought that we are rapidly moving into an era of Europe-wide drug licensing and that current European guidelines are rather limited in scope. In future, he believed that constraints would be placed on the use of licensed medicines because of financial and other reasons. Professor Hilary Calvert (University of Newcastle) agreed that the question of 'Is this agent clinically useful' was as important as 'Can it be safely administered'. This applied particularly to supportive agents such as colony-stimulating factors. He also felt that the CSM should have the power to insist that a requirement for further studies following granting of a phase II licence should be enforced.

Dr Martyn Evans (University of Wales) discussed the advent of the new system of Multicentre Research Ethics Committees (MREC) based on regions. There were a number of advantages to the new system including the inclusion of specified 'turnaround times' but also some disadvantages. In the new system, the Local Research Ethics Committees (LRECs) would be able to request minor local amendments to the patient information sheet or consent form but not the protocol. Nevertheless, an LREC may draw to the attention of an MREC Chairman serious concerns regarding the protocol, which that Chairman must consider and may refer the application back to the MREC for reconsideration. The LRECs' decision-making powers would be restricted to whether or not a trial should proceed locally. He felt strongly that the medical community should request a clearer 'spelling-out' of the local criteria upon which the LRECs would be able to base their judgement. Mrs Jennifer Blunt (Chair, North West MREC) thought that the old framework had not worked well. The problem was not with the standard of review but with the variability in the system. She pointed out the new system was not 'hierarchical' but that MRECs and LRECs would have separate and complementary duties. In the discussion, there was a strong view that specialization by MRECs might have been a better system. It was noted that the new system would be formally evaluated in 1999.

Dr Peter Brambleby (Consultant in Public Health, East Norfolk Health Authority) showed the distribution of expenditure of a typical health authority with the majority of spend on chronic disease, not acute specialities like oncology. NHS trusts usually recoup the cost of cancer drugs through prices for episodes of care in oncology. Even then, the total drugs budget of a typical district general hospital is around 5%. Appraisal of new drugs by health authorities follows a checklist which includes: evidence of clinically significant effect, guidelines for use including clinical responsibility, financial impact, added cost relative to added benefit, priority status relative to other demands, consultation and a final decision. A tension existed between the drive for local sensitivity and responsiveness in health care commissioning, which can introduce variation in availability of services, and simultaneous concern about widening inequalities in access across the country. He called on the help of the research community in the areas of epidemiology, effectiveness, economics, education and

*Received 14 April 1998*
*Accepted 22 April 1998*

ethics. Health authorities were busy places and the 'right decisions needed to be easy decisions'. Professor Stan Kaye (University of Glasgow) believed that setting funding priorities for new anti-cancer drugs could be very difficult. Oncologists should press for increased funding where a survival benefit is clear, but acknowledge the need to restrict usage to approved centres and/or clinicians using agreed guidelines. A particular problem arose when a drug gave clear clinical benefit in the absence of survival benefit. Quality of life measures were then extremely important and prospective randomized trials essential.

Professor Nick Thatcher (University of Manchester) analysed factors which disposed towards success or failure in cancer clinical trials. Successful trials were characterized by attractive protocol design, enthusiasm and commitment by a specific co-ordinator and the presence of adequate funding. Companies were increasingly reluctant to sponsor trials in the UK because of relatively high costs in comparison with other countries. Whereas, for example, in the United States and in mainland Europe, large amounts of money were being invested into trials of novel drug combinations in non-small-cell lung cancer, there were no such trials in the UK. Furthermore, the overall NHS budget for cytotoxic drugs is very low compared with that for other types of drugs. Professor Thatcher felt that there was no lack of innovative ideas for trials in the UK, but major bureaucratic hurdles and inadequate funding were major disincentives for progress. Professor David Kerr (University of Birmingham) emphasized the importance of establishing a strong clinical trials network within the country. He felt confident that there is the capacity within the UK for the carrying out of large, innovative phase III trials. The new system of central subventions for burdensome excess treatment costs should prove helpful. Setting of targets for cancer centres and units in terms of trial participation could be an important element of continued accreditation.

Professor Peter Selby (Clinical Director, Imperial Cancer Research Fund) emphasized the importance that should be attached to ensuring equality of access to all aspects of cancer medicine. He gave examples of dramatic inequalities which currently exist. Implementation of the Calman–Hine proposals was currently very 'patchy'. There is a clear need for improvements in the number of cancer specialists in the UK. Demographic changes over the next few years meant that the cost of implementing Calman–Hine would be approximately £35–40 million per year in 5 years' time. Dr John Toy (Director of Clinical Programmes, Cancer Research Campaign) emphasized that treatments can only be accessed by clinicians, and the harder clinicians pushed the more likely expensive treatments would be made available. He agreed that inequity of treatment was a major problem and that this was due to seemingly idiosyncratic clinical practice as well as financial constraints. There may need to be a greater emphasis on protocol-driven care based on findings that patients entered into clinical trials protocols tend to have better outcomes.

In conclusion, it was felt that the Workshop had been useful in highlighting a number of areas in which increased pressure from the cancer clinical community could be productive in improving access to new therapies.

Specific attention should be given to:

(a) Lobbying for the best available therapies to be made uniformly available to all subsections of the community.
(b) Participation in clinical trials to be a prerequisite for centres and units to become accredited and maintain their status under the Calman–Hine arrangement.
(c) Making strong bids whenever possible for money from the Lottery Opportunities Fund to support better cancer treatments and a uniformity of approach throughout the UK.

© Cancer Research Campaign 1999

